# Ross River Virus Provokes Differentially Expressed MicroRNA and RNA Interference Responses in *Aedes aegypti* Mosquitoes

**DOI:** 10.3390/v12070695

**Published:** 2020-06-27

**Authors:** James B. Sinclair, Sassan Asgari

**Affiliations:** Australian Infectious Disease Research Centre, School of Biological Sciences, The University of Queensland, Brisbane QLD 4072, Australia; james.sinclair@uq.edu.au

**Keywords:** alphaviruses, *Aedes aegypti*, Ross River virus, RNA interference, microRNA, piRNA

## Abstract

Alphaviruses are globally distributed and predominately transmitted by mosquitoes. *Aedes* species are common vectors for the clinically important alphaviruses—Chikungunya, Sindbis, and Ross River (RRV) viruses—with *Aedes aegypti* also being a vector for the flaviviruses dengue, Yellow Fever, and Zika viruses. *Ae. aegypti* was putatively implicated in the large 1979–1980 South Pacific Islands outbreak of RRV—the leading cause of arboviral disease in Australia today. The RNA interference (RNAi) defense response in mosquitoes involves a number of small RNAs, with their kinetics induced by alphaviruses being poorly understood, particularly at the tissue level. We compared the small RNA profiles between RRV-infected and non-infected *Ae. aegypti* midgut and fat body tissues at 2, 6, and 12 days post-inoculation (dpi). RRV induced an incremental RNAi response, yielding short interfering and P-element-induced-wimpy-testis (PIWI)-interacting RNAs. Fourteen host microRNAs were differentially expressed due to RRV with the majority in the fat body at 2 dpi. The largely congruent pattern of microRNA regulation with previous reports for alphaviruses and divergence from those for flaviviruses suggests a degree of conservation, whereas patterns of microRNA expression unique to this study provide novel insights into the tissue-specific host-virus attributes of *Ae. aegypti* responses to this previously unexplored old-world alphavirus.

## 1. Introduction

Members of the *Alphavirus* genus (*Togaviridae*) are mosquito-borne enveloped RNA viruses that are globally distributed, and those of medical significance are attributed to causing encephalitic (e.g., Eastern (EEEV), Western (WEEV), and Venezuelan Equine Encephalitis (VEEV) viruses) or arthritogenic (e.g., Ross River virus (RRV), Chikungunya virus (CHIKV), and the archetypal Sindbis virus (SINV)) diseases [1]. Alphaviruses are maintained through propagation in sylvatic transmission cycles (animal–mosquito–animal) between amplifying vertebrate hosts in which the titer of viremia is high and sustained [2]. Occasionally, transmission cycles encroach into urban environments via short-term amplifying hosts such as peridomestic animals (e.g., bats, birds, cats, dogs, or possums), however, during epidemics, transmission cycles that are entirely urban (human–mosquito–human) can occur in densely populated regions [3]. 

RRV is an arthritogenic virus endemic to Australia, Papua New Guinea, and South Pacific Islands [4,5,6]. The virus causes epidemic polyarthritis (EPA; otherwise known as Ross River disease or fever) and is the leading cause of viral disease in Australia with a rolling annual mean between 2012 and 2017 exceeding 6000 cases [7]. On the basis of previous estimates and factoring in inflation, the national cost of EPA in 2018 exceeded AUD 9.3 million in medical expenses, whereas the true magnitude of associated costs is unclear when also factoring in mosquito surveillance, control, and response activities (e.g., vector control exceeds AUD 13 million per annum), or outbreak impact on tourism and industry [8,9].

RRV was first isolated from *Aedes vigilax* near Townsville, Queensland, in 1959 [10]. In Australia, *Ae. vigilax* is an epidemiologically important saltmarsh mosquito in the coastal regions of the north [11]. Other important species of mosquitoes include *Aedes camptorhynchus* in the south [4], the inland freshwater species *Culex annulirostris* [11], and *Aedes notoscriptus* in urbanized regions [12]. Interestingly, *Ae. notoscriptus* is associated with transmission cycles of RRV involving the Australian brushtail possum [13,14], both of which are prevalent in New Zealand [15,16]. Although there are no records of RRV circulation in New Zealand, the presence of *Ae. notoscriptus* and brushtail possums indicates a significant potential for local transmission if RRV were to be introduced [17]. There is no definitive record of the species of mosquitoes involved in the Pacific Islands epidemic of 1979–1980, whereas those implicated on the basis of prevalence and capacity to readily transmit RRV included *Ae. vigilax*, *Cx. annulirostris*, *Aedes polynesiensis*, *Aedes oceanicus*, *Culex quinquefasciatus*, and *Aedes aegypti* [18,19]. RRV has been isolated from 42 species of mosquitoes [4,11,20], whereas laboratory competence studies have only indicted 10 as being capable of transmission [4]. RRV has never been isolated from *Ae. aegypti* in the field, and this species of mosquito is not considered a vector of RRV; however, multiple competency studies have shown that *Ae. aegypti* can readily transmit the virus (e.g. [21]).

*Ae. aegypti* is unrivalled in medical importance, being culpable for our most impactful diseases including CHIKV, dengue virus (DENV), Yellow Fever virus (YFV), and Zika virus (ZIKV), wherein, collectively, tens of millions of cases of disease and tens of thousands of deaths occur globally each year [22,23,24,25]. The medical importance of *Ae. aegypti* is underscored by its capacity to adapt to a range of ecological niches, both spatial and temporal, to transmit multiple disparately related and highly pathogenic viruses, and for its natural drive to preferentially seek out human blood. The capacity of *Ae. aegypti* to vector a wide range of viruses and its ability to adapt to foreign environments stresses our need to better understand the molecular host–virus interactions in this mosquito.

Genomic studies analyzing differentially regulated genes have enabled significant progress in elucidating mosquito innate immune responses to arbovirus infection. Sim et al. (2005) pioneered this approach, reporting the modulation of *Anopheles gambiae* gene expression in response to O’nyong-nyong virus (ONNV: *Alphavirus*) infection, and later in 2007 they demonstrated that this modulation resulted in the production of heat shock protein cognate 70B (HSC70B), which impeded ONNV replication [26,27]. In response to arboviral infection, *Ae. aegypti* has been reported to initiate Toll [28], c-Jun N-terminal kinase (JNK) [29], immune deficiency (Imd) [30], and Janus kinase-signal transducer and activator of transcription (JAK-STAT) pathways [31]. The JAK/STAT pathway has been reported to be utilized by *Ae. aegypti* to inhibit DENV [32] but not CHIKV [31,32], whereas there are contradictory reports for ZIKV [32,33].

RNA interference (RNAi) has been recognized as an important mechanism by which eukaryotes regulate transcriptional and post-transcriptional gene expression, mRNA methylation, and chromatin remodeling [34]. The RNAi pathway produces small non-coding RNAs (sncRNAs: ≈20–30 nt) that are involved in a range of biological processes including host–pathogen interactions and anti-viral responses [34,35]. In insects, the major sncRNAs are short interfering RNA (siRNA), P-element-induced-wimpy-testis (PIWI)-interacting RNA (piRNA), and microRNA (miRNA).

Campbell et al. (2008) showed that *Ae. aegypti* produced complementary siRNAs in response to a SINV challenge and that silencing Argonaut 2 (Ago2) or Dicer-2 (Dcr2) endonucleases caused a transient increase in SINV replication [36]. Concomitantly, Cirimotich et al. (2009) demonstrated that Flock House virus (FHV: *Nodaviridae*, genus *Alphanodavirus*) B2 protein inhibited the siRNA response by shielding SINV double stranded RNA (dsRNA) from RNAi detection, resulting in a fatal viraemia in *Ae. aegypti* [37]. Additionally, *Ae. aegypti* has been shown to use siRNA to control DENV [38] and CHIKV [31], and in *Ae. aegypti*-derived Aag2 cells, the Semliki Forest virus (SFV: *Togaviridae*, genus *Alphavirus*) [39]—examples that underscore the versatility of the RNAi response.

Several studies have detected alphavirus-derived piRNAs in mosquitoes infected with SFV, CHIKV, ONNV, and SINV, although the consequence of these piRNA responses remain unclear [40]. In *Ae. aegypti*, only 19% of total piRNAs are directed against transposons [41], whereas a substantial portion of genes involved in germline and embryonic development in *An. gambiae* encode piRNA transcripts, suggesting that piRNAs in mosquitoes have multiple functions [34]. 

The ingestion of viremic blood by a mosquito results in a diverse and complex miRNA response [42]. Liu et al. (2015) showed that miR-1174 and miR-1175 were upregulated in the gut of *Ae. aegypti* following a blood meal [43]. Bryant et al. (2010) showed that miR-8, miR-14, and miR-275 were upregulated in the fat body of *Ae. aegypti* following a blood meal and that these are involved in digestion and egg development [44]. Campbell et al. (2014) reported 35 differentially abundant miRNAs in *Ae. aegypti* in response to DENV2 infection, with the majority of predicted targets involved in transport, signal transduction, and metabolism [45]. In the saliva of *Ae. aegypti* infected with CHIKV, Maharaj et al. (2015) identified five miRNAs that were predicted to be involved in the regulation of CHIKV infection [46]. 

There are very few studies demonstrating miRNA responses in mosquitoes infected with RNA viruses at the tissue level. In particular, there are no studies on RRV. This may be because none of the important vectors of RRV have been sequenced and therefore a platform for comparative genomics to an epidemiologically relevant reference cannot be made. The genome of *Culex quinquefasciatus* has been sequenced [47], although vector competence studies have shown this mosquito to be poorly susceptible to RRV infection and, therefore highly unlikely to be involved in RRV maintenance [48]. *Ae. aegypti* has been shown to competently transmit and potentially facilitate the global spread of a range of viruses, including RRV, which stresses our need to better understand the repertoire of molecular host–virus interactions in this mosquito. Further, the complete genome, transcriptome, and miRNAome of *Ae. aegypti* are well characterized. In this study, we revealed the differentially abundant small RNA profile of the pertinent organs (midgut and fat body) in *Ae. aegypti* infected with RRV. These results provide an important and relevant contribution to our understanding of the molecular host–virus interactions within a medically significant vector infected with an old-world alphavirus.

## 2. Materials and Methods

### 2.1. Ethics Statement

The T48 strain of RRV used in this study was kindly provided by Jody Peters of the School of Chemistry and Molecular Biosciences, The University of Queensland. The virus was originally isolated from *Ae. vigilax* mosquitoes (Townsville, QLD, Australia). RRV protocols were approved by the University of Queensland Biological Sciences Biosafety Committee (Reference number: IBC/231B/SBS approved on 22 May 2018).

### 2.2. Mosquito Infections with Ross River Virus

*Ae. aegypti* (Innisfail strain) mosquitoes were propagated from eggs and raised in an insectary maintained at 27 °C, 80% relative humidity, and a 12 h photoperiod. RRV (T48 strain) was amplified in C6/36 cells (*Ae. albopictus*) cultured at 28 °C in a 1:1 *v*/*v* mixture of Schneider’s Drosophila Medium (Life Technologies, California, USA) and Mitsuhashi and Maramorosch (Himedia, Mumbai, India) medium supplemented with 2% fetal bovine serum (FBS). The virus was then amplified in African green monkey kidney (Vero) cells cultured at 37 °C in Opti-MEM (Life Technologies, California, USA) media with 2% FBS for 48 h. Control C6/36 and Vero cells that did not include virus were cultured each time and in the same way. The same volume of C6/36 cell supernatant that was used to inoculate Vero cells was also transferred between control cultures. Virus amplified in Vero cells was mixed 1:1 v/v with whole human blood (Australian Red Cross) and 0.1% ATP (phagostimulant). Females aged 4–6 days old were fed blood mixtures with or without virus at 2 × 10^5^ plaque forming units per mL (pfu/mL). At 2, 6, and 12 days post-feeding (dpf), mosquitoes were cold anaesthetized and transferred to −80 °C storage until use.

### 2.3. Mosquito Dissection and RNA Extraction

The midgut and fat body tissues were dissected from a total of 180 mosquitoes yielding 360 organs. The organs were pooled in lots of 10 yielding 36 samples comprising the tissues fat body (*N* = 18) or midgut (*N* = 18) that were infected (*N* = 9) or uninfected (*N* = 9) at day 2 (*N* = 3), day 6 (*N* = 3), or day 12 (*N* = 3). Total RNA was extracted from samples (*N* = 36) using the miRNeasy Mini Kit following the manufacturer’s protocol (Qiagen, Hilden, Germany). For practical reasons, we did not confirm infection or non-infection in all mosquitoes (*N* = 180). During dissection, the RNA from the carcass remnants of 10 randomly selected mosquitoes sampled from each day and treatment was used to detect virus presence or absence (control) by reverse transcription quantitative PCR (RT-qPCR) on a Rotor-Gene Q (Qiagen, Hilden, Germany) machine using *ribosomal protein subunit 17* (RPS17) and in-house-designed RRV-specific primers (Table S1). Mean normalized expression (MNE) of samples relative to RPS17 was calculated using the second derivative maximum take-off and amplification efficiency values produced by the Rotor-Gene Q software. 

### 2.4. Library Preparations and Sequencing

The TruSeq Small RNA protocol (Illumina) was used to generate and sequence complementary DNA (cDNA) libraries from small-input RNA on a short-read sequencing platform with an Illumina Hi-Seq machine (Illumina). Briefly, cDNA libraries were amplified from 1 µg of size-fractionated total RNA ligated with indexed 3’ and 5’ adapters (Table S1). Libraries were size-selected by polyacrylamide gel, purified, and concentrated. An Agilent Technologies 2100 Bioanalyzer (California, USA) was used to assess purity for quality control. Illumina RTA software (1.17.21.3) was used for base-calling for quality control, and Illumina bcl2fastq 2.17 software was used for demultiplexing. Final raw reads were in FASTQ format with the 3’ adapter retained. The raw data for infected and uninfected *Ae. aegypti* samples generated in this study were deposited in the Sequence Read Archive (SRA) under the accession PRJNA635740.

### 2.5. Small RNA Analysis

The software tool Fastq_quality_filter (FASTX-toolkit) (v0.0.6) [49] was used to earmark raw reads that had at least 95% of their sequence content with a phred quality score above 20. Using Flexbar [50], we trimmed the 3’ adaptors (Table S1), allowing a minimum overlap of 4 nucleotides (nt), and remaining reads with less than 16 nt were discarded. The length distribution of reads was visualized using scales [51], plyr [52], and ggplot2 [53] in the R package (v3.6). The miRPro pipeline [54] was used to enumerate and categorize the samples as miRNA content and other RNA biotypes. Cleaned reads were mapped to the most recent miRBase repository (v21) [55] using Novoalign (Novocraft, Selangor, Malaysia) and both mature and novel mature miRNAs were enumerated with htseq-count (HTSEQ 1.11) [56] and Samtools algorithm [57]. Remaining reads were mapped to the most current *Ae. aegypti* genome (AaegL5; VectorBase) [58] for RNA reads categorization. Differential expression of miRNAs (Table S2) was calculated from concatenated mature and novel mature miRNA counts using DESeq2 (v3.11) [59] following various namesake Bioconductor vignettes in the R package [60,61]. The counts from all samples were included in the experimental design for differential expression, which included the three main factors (1) tissue (tsu), (2) time (day), and (3) condition (cnd) and interaction terms between these factors (design = tsu + tsu:day + tsu:day:cnd). The levels for each factor were (1) tsu: fat body (fb) and midgut (mg); (2) day: 2, 6, and 12; and (3) cnd: control (con) and infected (rrv). Fat body, day 2, and control was set as the reference levels by which change was compared. The coefficients derived from the design enabled comparison between tissues mg and fb irrespective of infection (tsu_mg_vs_fb), the effect of age between days with reference to deviance from day 2 (reference) irrespective of infection (e.g., tsu.fb.day6 or tsu.mg.day12), the effect size of condition within tissues within day (e.g., tsufb.day2.cndrrv), within tissue between days (e.g., contrast = tsufb.day2.cndrrv and tsufb.day6.cndrrv), or between tissues within day (e.g., contrast = tsufb.day2.cndrrv and tsumg.day2.cndrrv). The effect size of condition between tissues between days (e.g., tsufb.day2.cndrrv and tsumg.day12.cndrrv) was not explored. To test for significance, we used the Wald test (alpha, 0.05) for the pairwise statistical comparison of probability calculated between the coefficients generated by our experimental design. The threshold for significance was set at a false discovery rate (FDR) of 0.05. The FDR implemented in DESeq2 is an adjusted *p*-value given by the Benjamini and Hochberg algorithm [62]. We used the approximate posterior estimation for generalized linear model (apeglm) function [63] to exclude results from low or highly variable counts and further excluded results with a baseMean < 100. In DESeq2, the term baseMean refers to the mean normalized count across all samples. Remaining miRNAs were considered significantly differentially expressed (SDE). In summary, our criteria qualifying miRNA significance were for a normalized mean count of samples higher than 100, a FDR less than 0.05, and an effect size fold change (FC) of 1.5.

### 2.6. RNAi Activity Analysis

For siRNA and piRNA identification, reads that did not align with either the *Ae. aegypti* genome (AaegL5) or miRBase records were mapped to the genome of the T48 strain of RRV (GenBank: GQ433359.1) [64] using Novoalign (Novocraft, Selangor, Malaysia). BAM alignment files were filtered by strand and lengths of 21 nt or 27–29 nt for siRNA and piRNA reads analyses, respectively. We used kpPlotBAMCoverage in karyoploteR (v1.14.0) within the R package (v3.6) [65] to visualize the genome coverage of siRNA and piRNA reads. We used WebLogo software (v3) [66] to visualize U_1_ and A_10_ piRNA signatures, and 10 nt corroborative overlap signatures were assessed and scored using the public Galaxy server at https://mississippi.sorbonne-universite.fr [67]. 

### 2.7. The miRNA Target Identification

The algorithms miRanda [68], RNAhybrid [69], TargetScan [70], PITA [71], and RNA22 [72] were used to predict miRNA targets against *Ae. aegypti* 3’UTR sequences. For this, a bed file of headers containing the phrase three_prime_utr and corresponding coordinates was extracted from an annotation file (Aedes-aegypti-LVP_AGWG_BASEFEATURES_AaegL5.2.gtf) with grep and used as a template for BEDTools [73] to extract those corresponding 3’UTR sequences from file (Aedes-aegypti-LVP_AGWG_CHROMOSOMES_AaegL5.fa). Sequences shorter than 30 nt were discarded, and to remove duplicates for genes with splice variants, we used only the longest 3’UTR. All target prediction programs were operated locally on a Linux machine using default command line parameters. We referred to the results exclusion criteria used by Oliveira et al. (2017) to guide the exclusion criteria that we used in this study. For miRanda, we used a minimum score of 150 and a minimum folding energy (mfe) of < −18 (kcal/mol) [74]. For RNA22, we used a *p*-value of < 0.1 and mfe values < −12 (kcal/mol). For RNAhybrid, we set the *p*-value at 0.05 and mfe values < −20 (kcal/mol). For PITA, we used mfe values < −18 and excluded 6mer types from both PITA and TargetScan predictions. The intersect of targets predicted by at least three programs were used for downstream analysis (Table S3).

### 2.8. Gene Ontology

The Module Functional Analysis in OmicsBox (Biobam, Valencia, Spain) was used for gene ontology (GO) annotation and analysis (Table S4) of the miRNA targets predicted by at least three programs [75]. This module used National Centre for Biotechnology Information (NCBI) Blast against homologous *Ae. aegypti* sequences to establish the protein identity of the query sequences from this study. The corresponding GO IDs and GO names of the mapped and annotated proteins were retrieved using OmicsBox InterPro scan and these were linked with Kyoto Encyclopedia of Genes and Genomes (KEGG) pathways (Table S5). The statistics and graphical representation of all the results was produced by the OmicsBox program.

### 2.9. RT-qPCR Analysis

The miScript II RT kit (Qiagen, Hilden, Germany) was used following the manufacturer’s protocol to synthesize cDNA from 500 ng RNA per 20 µL reaction using the 5× HiSpec Buffer and the proprietary-sequence universal primer on a Veriti thermal cycler (Applied Biosystems, California, USA). cDNA samples were diluted in 200 µL nuclease-free water and 2 µL thereafter amplified in 10 µL duplicate reactions with miRNA-specific primers (Integrated DNA Technologies, Iowa, USA) by RT-qPCR using miScript SYBR Green PCR kit reagents (Qiagen, Hilden, Germany) on a 72-well Rotor-Gene Q thermocycler (Qiagen). The thermocycling parameters used for amplification were 95 °C for 15 min, followed by 40 cycles of 94 °C for 15 s, 60 °C for 30 s, and 70 °C for 30 s. The mean normalized expression (MNE) of samples was calculated using the second derivative maximum and corresponding amplification efficiencies generated by the Rotor-Gene Q software [76]. The *Ae. aegypti* small nuclear RNA U6 was used as the calibrator. The log_2_ ratio between control and treatment MNE values were used to calculate fold change. Three biological replicates were used per treatment. 

## 3. Results and Discussion

### 3.1. Illumina Sequencing of Small RNAs

**Figure 1:**
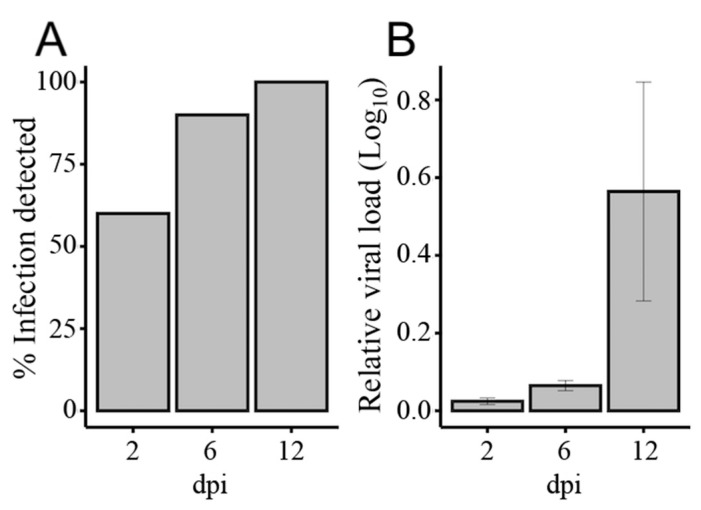
Measurement of Ross River virus (RRV) infection in *Aedes aegypti* mosquitoes at 2, 6, and 12 days post-infection (dpi) by RT-qPCR. (**A**) Rate of infection: percentage of mosquitoes with a detectable degree of RRV infection (*N* = 10), and (**B**) their relative viral load (*N* = 6). Error bars indicate the standard error of the mean of normalized expression values. The *Ae. aegypti* ribosomal protein subunit 17, *RPS17*, gene was used as the internal calibrator.

An Illumina sequencing platform was used to produce small RNA profiles of RRV-infected and non-infected *Ae. aegypti* mosquitoes. To investigate host miRNA and RNAi responses to RRV infection, we extracted RNA samples from midgut and fat body tissues of mosquitoes collected at 2, 6, and 12 days post-infection (dpi). The rates of viral infection were determined from a subset of individual mosquitoes (*N* = 10) from days 2, 6, and 12 and the relative viral load in those mosquitoes with a detectable degree of RRV infection (*N* = 6) was confirmed by RT-qPCR, which demonstrated a progressive increase in viral load over time (Figure 1). No virus was detected in uninfected negative control mosquitoes. Considering that carcasses were used for determination of virus infection after dissection of tissues, 100% dissemination of virus at 12 dpi suggested high rate of infection even if it was detected in about 50% of mosquito carcasses at 2 dpi when virus was at early stages of infection in the midgut, which was already removed from mosquito bodies.

**Figure 2:**
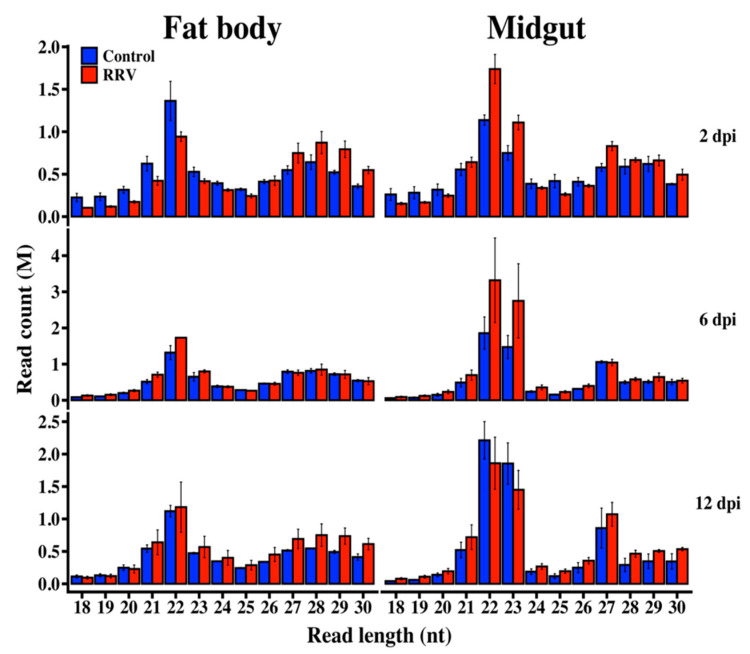
Length distribution of quality-filtered and adapter trimmed reads from *Ae. aegypti* fat body and midgut tissues at 2, 6, and 12 dpi. Error bars indicate the standard error of the mean of biological replicates (*N* = 3).

The combined yield of raw data obtained from control and infected small RNA libraries was 200 and 232 million reads, respectively (Table S2). Of the reads, we discarded 6–28% in different libraries due to their low-quality score or lack of adapter sequence. A total of 94% of the annotated *Ae. aegypti* miRNAs referenced by miRBase were found among all the samples in our data, representing 7–22% of clean reads in different libraries. The sequence length distributions in all libraries expressed peaks at 21–23 nt, consistent with the characteristic lengths of miRNA and short interfering RNA (siRNA), and peaks at 27–29 nt representing piRNA—a common feature of insect small RNA libraries (Figure 2).

### 3.2. Differential Expression of Ae. aegypti miRNAs in Response to RRV Infection

**Figure 3:**
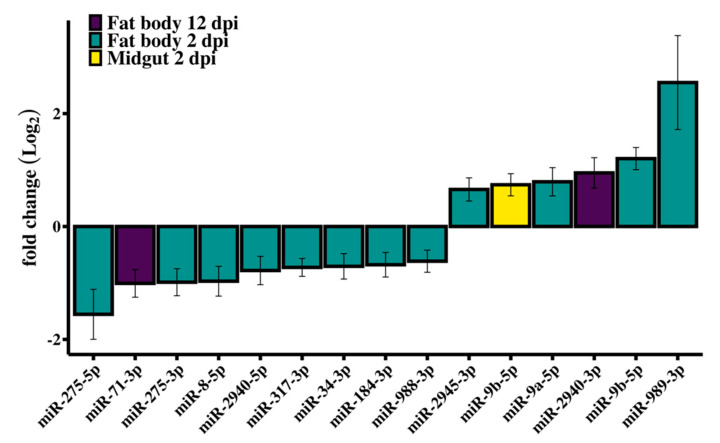
RRV caused differential expression of *Ae. aegypti* miRNAs. Log_2_ fold changes of miRNA expression following infection with RRV at 2 dpi in fat body (green) and midgut (yellow) and in fat body at 12 dpi (purple). Error bars represent the standard error of the mean of biological replicates (*N* = 3).

**Figure 4:**
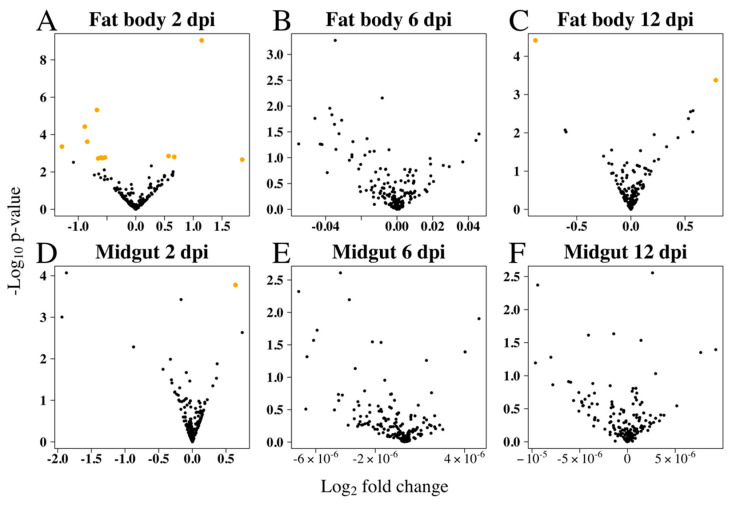
RRV caused differential expression of *Ae. aegypti* miRNAs mostly early in infection. Volcano plots show miRNA log_2_ fold changes due to RRV infection in fat body at (**A**) 2 dpi, (**B**) 6 dpi, and (**C**) 12 dpi, and in midgut at (**D**) 2 dpi, (**E**) 6 dpi, and (**F**) 12 dpi. Significantly differentially expressed miRNAs (orange) were found in fat body samples at 2 dpi and 12 dpi and in midgut samples at 2 dpi.

Small RNA library analysis of RRV-infected *Ae. aegypti* fat body and midgut tissues identified 14 SDE miRNAs at different time points compared with uninfected mosquitoes (Figure 3). Interestingly, miRNA modulation was prominent early in infection at 2 dpi, and by day 12, when the viral load was higher, most miRNAs in both tissues were stably expressed (Figure 4). In contrast to siRNAs, there is very little evidence that miRNAs directly target viral genomes [77], and therefore we would not expect to see the positive correlation of changes in miRNA expression with viral load. It is then perhaps not surprising to see the dramatic shift in miRNA expression during the early adjustment phase to infection when the greatest number of changes in target gene expression occur. The identified miRNAs were predominately downregulated (60%) and from the fat body (93%) tissue (Table 1). An intriguing observation is the preponderance for the downregulation of *Ae. aegypti* miRNAs reported for both flavivirus (DENV and ZIKV) [45,78] and alphavirus (CHIKV and SINV) [46,79,80] infections, although direct comparison between these studies and our results is difficult considering the different time points or samples used (whole mosquito/tissues/cell line). Myles et al. (2008) reported an overall depletion of miRNA expression in *Ae. aegypti* infected with SINV, which was the first record in the literature investigating the miRNA expression of *Ae. aegypti* infected with an old-world alphavirus, when *Ae. aegypti* miRNAs had not yet been annotated and the authors’ analysis for differential expression used *Drosophila melanogaster* miRNAs as reference [80].

**Figure 5:**
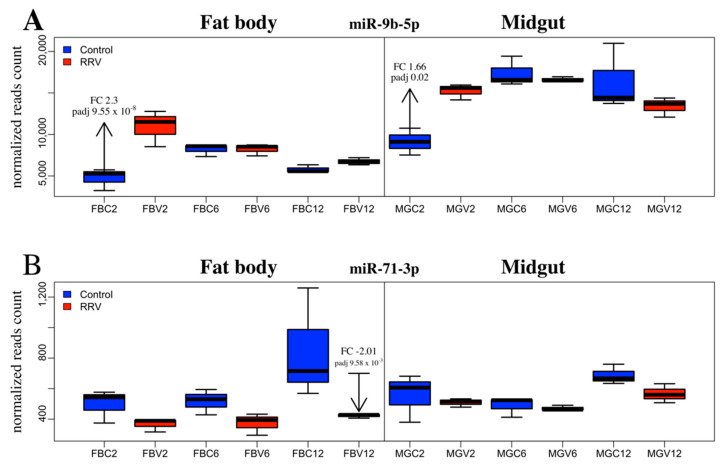
*Ae. aegypti* miRNA differential expression due to RRV infection between tissues and across time. The expression profile of (**A**) miR-9b-5p and (**B**) miR-71-3p, in control (blue) and infected (red) fat body and midgut samples at 2, 6, and 12 dpi. Significant changes are labelled with the fold change (FC) and adjusted *p*-value (padj). The boxplot and whiskers show the mean of the normalized reads counts of the biological replicates (*N* = 3).

miR-9b-5p was upregulated in the midgut (FC 1.67) at 2 dpi and was the only SDE miRNA found in midgut samples (Figure 5A). This miRNA was also upregulated in the fat body at 2 dpi (FC 2.29), which was the most significant result from this study (FDR, 9.55e-08). Maharaj et al. (2015) reported downregulation of this miRNA from a read count of 64 to 34 in the saliva of *Ae. aegypti* infected with CHIKV at 10 dpi, although the change in miR-9b expression was not considered statistically significant [46]. By contrast, in *Ae. albopictus*, Liu et al. (2015) found miR-9b to be significantly downregulated at 7 dpi with DENV. Considering about 50% virus dissemination was detectable in mosquitoes at 2 dpi (Figure 1A), we speculate that the early changes in fat body miRNAs could be part of an early systemic response to virus infection following the receipt of cytokines from infected midgut cells via hemolymph. Crosstalk between tissues for immune induction following blood feeding has been documented (reviewed in [81]). In contrast to the significant downregulation of miR-2940-3p in the study by Liu et al. (2015), in our study, miR-2940-3p was upregulated. By comparison, miR-184-3p and miR-2940-5p were significantly downregulated in Liu et al. (2015) [82] and these miRNAs were also downregulated in our study. The continued enquiry of miRNA profile changes elicited by disparate pathogens in this mosquito or other dipterans is required to better clarify their evolutionary conservation and functional significance in response to infection.

The most abundant SDE miRNAs found in this study—miR-184-3p, miR-275-5p, miR-2940-5p, miR-317-3p, and miR-989-3p—are commonly reported as highly abundant and widely conserved [83]. Additionally, miR-989-3p (FC 5.85) and miR-275-5p (FC 0.34), which were both from fat body tissue at 2 dpi, had the most extreme fold changes. By comparison, in *An. gambiae* infected with *Plasmodium bergei*, miR-989 was also significantly upregulated (FC 4) and deemed exclusive to midgut tissue [84], whereas other studies had contended that, with reference to results comparing the conserved homologues in both *Anopheles stephensi* and *Ae. aegypti*, miR-989-3p is predominantly expressed in ovaries [85], and rarely in the midgut tissue [86]. We detected miR-989-3p in the midgut tissue at 2 dpi, whereas its change in expression compared to uninfected samples was not statistically significant. Other studies reporting the significant differential expression of miR-989-3p involved the vectors *Ae. albopictus* and *Culex quinquefasciatus* infected with West Nile virus (WNV) [87], *Ae. aegypti* infected with DENV [88] or ZIKV [78], or *Ae. aegypti* cells (Aag2) infected with *Wolbachia* [89]. According to our criteria for significance, there were no SDE miRNAs detected from mosquitoes at 6 dpi in either tissue.

The only SDE miRNAs from day 12 mosquitoes were miR-71-3p and miR-2940-3p in the fat body tissue. An analysis of the change in expression of miR-71-3p revealed that in control mosquitoes this miRNA became progressively more abundant throughout the time course, whereas in the fat body at 12 dpi, miR-71-3p was significantly downregulated. By comparing samples across time, it appeared that the condition effect due to RRV was an impediment to the natural age-related upregulation of miR-71-3p by day 12 (Figure 5B). It would be interesting to investigate the target genes of miR-71-3p, the consequence of their not being silenced at that age, and whether their continued expression was part of a deliberate anti-viral defense response or caused by the virus. 

Saldaña et al. (2017) also noted a disproportionately low number of SDE miRNAs in *Ae. aegypti* mosquitoes infected with ZIKV, despite the pronounced viral load reached by day 14 [78]. Comparing results for miR-2940-3p from this study and Saldaña et al. (2017), the miRNA shows remarkably similar measures of abundance and degree of upregulation (FC ≈ 2). While whole *Ae. aegypti* mosquitoes were infected with a flavivirus and expression had peaked earlier at day 7 in Saldaña et al. (2017), the measures of high abundance are consistent with published results for this miRNA from both flaviviruses [78,82] and alphaviruses [79]. *Wolbachia* is an endosymbiotic bacterium that is currently used to control DENV spread as its presence in *Ae. aegypti* blocks replication of a number of viruses [90]. In *Ae. aegypti*, *Wolbachia* induces and utilizes miR-2940 to suppress the expression of pelo protein [91], upregulate host metalloprotease f41 ftsh transcripts [89], and downregulate the expression of *Ae. aegypti* DNA methyltransferase 2 [92], activities that aid its preservation in the host and contribute to inhibition of replication and subsequent transmission of co-infected DENV. 

Mosquito miRNA induction by CHIKV has been studied extensively, however, research into other alphaviruses is needed to supplement the literature for comparing between species to further develop a characterization of their distinct molecular responses and elucidate with greater clarity the functional reasons for miRNA involvement and why conservation or divergence in miRNA form and function has evolved. Because this project is a foray into the *Ae. aegypti* miRNA responses to an old-world alphavirus, we prioritized our analysis of miRNA function to those also published with reference to CHIKV or *Ae. aegypti* fat body.

### 3.3. RT-qPCR Validation of Differentially Expressed miRNAs

**Figure 6:**
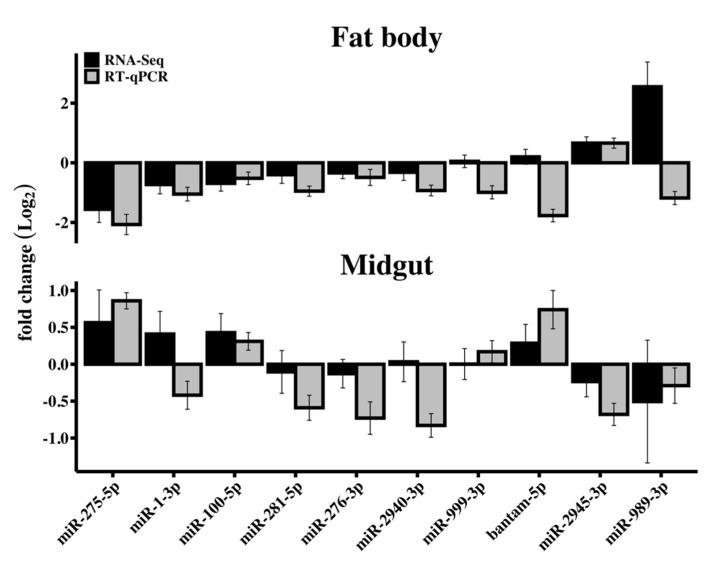
RRV induced differential expression of *Ae. aegypti* miRNAs. Contrasting RNA-Seq and RT-qPCR measurement of RRV-induced differential expression of 10 miRNAs in *Ae. aegypti* fat body and midgut RNA samples at 2 dpi. Error bars represent the standard error of the mean of the biological replicates (*N* = 3).

The relative expression of 10 miRNAs was ascertained by RT-qPCR from RNA samples extracted from fat body and midgut tissues from control and RRV-infected samples at 2 dpi to validate the RNA-Seq data and subsequent assessment of differential expression of miRNAs (Figure 6). The 10 miRNA candidates for this experiment were selected to represent the scope of fold changes determined by DESeq2, which included those showing the most (miR-989-3p) and least (miR-999-3p) divergence. The direction of change was mostly consistent between methods with discrepancies for miR-1-3p and miR-2940-3p from midgut and miR-999-3p, bantam-5p, and miR-989-3p from fat body, which notably also showed the largest discrepancy between the reported magnitudes of fold change.

### 3.4. The miRNA Target Analysis

**Figure 7:**
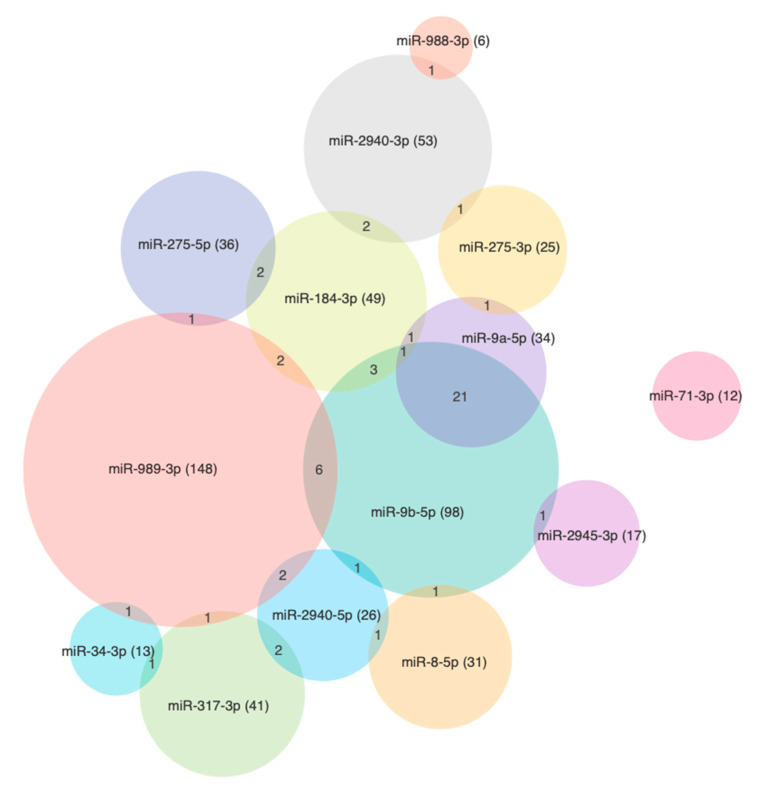
Venn diagram showing the number of predicted targets for each miRNA, wherein the intersect shows the number of predicted targets in common.

The putative *Ae. aegypti* 3’UTR mRNA targets of the SDE miRNAs determined in this study (Table 1) were predicted using the default parameters of miRanda, RNAhybrid, TargetScan, RNA22, and PITA software. From the intersect of targets predicted by at least four programs, we found 788 genes as putative binding sites for at least one miRNA. Among those, 65 genes were associated with at least 2 miRNAs, 6 genes with 3 miRNAs, and AAEL003953 (LOC5579800, ec:2.7.1.123) was found to be a putative target for miR-9b-5p, miR-275-5p, miR-2940-3p, and miR-2940-5p (Figure 7). The greatest number of putative binding sites were for miR-989-3p (367), miR-9b-5p (287), and miR-2940-3p (73), which were also determined in this study to have the highest upregulation and by the same rank (Table 1).

**Figure 8:**
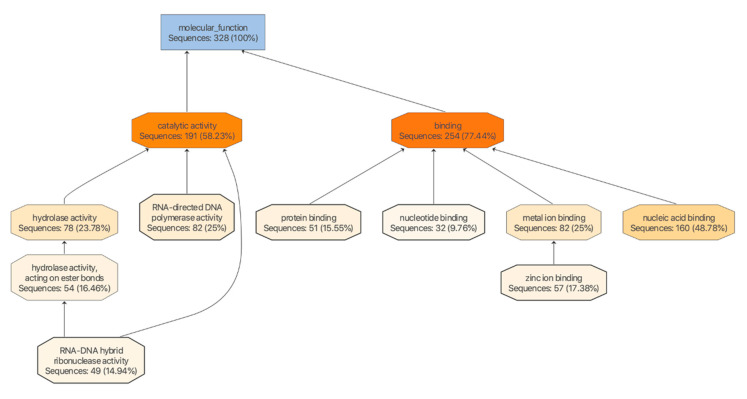
Molecular function. Tree map of gene ontology descriptions ascribed to genes from this study showing the interrelationship between the parent (blue) and children (shades of orange) ontologies involved in the molecular functions of binding and catalytic activities.

**Figure 9:**
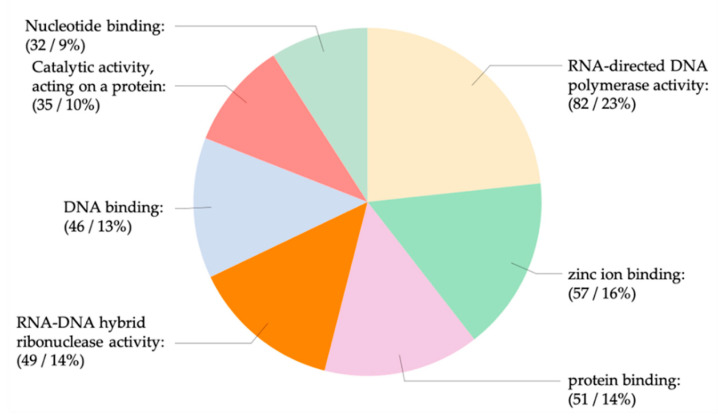
Molecular function. The number and percentage of genes from this study ascribed to various molecular functions.

An analysis of the KEGG pathways revealed a number of binding sites that were for genes putatively involved in mosquito immunity and signaling (Table S5). There were 328 sequences with the molecular function annotation (GO:0003674) comprising genes ascribed to hydrolase (78), polymerase (82), and iron binding (57) activities (Figure 8 and Figure 9). Both miR-989-3p and miR-9b-5p were associated with genes involved in zinc-finger protein and the mitogen-activated protein kinase (MAPK) signaling pathway as described by the response to stress ontology (GO:0006350). It has been shown that the expression of zinc-finger antiviral protein inhibits the replication of members of the *Alphavirus* genus including SINV, SFV, VEEV, and RRV [93]. AAEL013596 is a phosphatidylinositol 3-kinase (PI3K) regulatory subunit and is involved in the Toll pathway. In *Drosophila*, the Toll-7 transmembrane receptor activates antiviral autophagy facilitated by the phosphatidylinositol-3-kinase and the mammalian target of rapamycin (PI3K-Akt-mTOR) signalling pathway [94], whereas for SINV, which forms membrane bound replication complexes, the activation of this pathway in *Drosophila* becomes pro-viral, facilitating its cap-dependent viral RNA translation [95]. In human cells, the metabolic changes induced by the hyperactivation of PI3K-Akt by SFV and RRV is also pro-viral [96].

The beta subunit kinase, AAEL003245, is an inhibitor of nuclear factor kappa B and involved in the mTOR signaling pathway, which relates to vitellogenesis, protein synthesis, and motility. Genes were associated with the Wingless signaling pathway (Wnt), which works synergistically with the mTOR pathway in regulating vitellogenesis [97]. Following a blood meal, miR-8 is significantly upregulated in *Ae. aegypti* fat body and was shown to target the Wnt pathway to promote the production of yolk protein precursors (YPP) and lipid accumulation used for vitellogenesis [98]. Conversely, in this study, miR-8 was significantly downregulated in the fat body of RRV-infected *Ae. aegypti* mosquitoes, which may signify an active response to stall vitellogenesis. Schwenke et al. (2016) showed that pleiotropic signaling mechanisms regulate the allocation of metabolic resources between reproductive and immune processes in female insects, and that egg production is stopped to reallocate energy resources to the defense response during infection [99]. In humans, elements of the mTOR signaling pathway are modulated by the Influenza A virus [100] and ZIKV [101], which promote viral replication during late stage cell stress.

To corroborate our findings with published evidence, we compared our list of predicted miRNA target genes with studies involving changes in the *Ae. aegypti* transcriptome due to arbovirus infection. We only included transcripts that were reported as significantly modulated and predicted as a target for at least one miRNA by no less than three algorithms. In total, we found 18 unique genes, representing the predicted targets for 12 out of the 14 miRNAs from our study, which were shown to be significantly modulated in different studies involving whole *Ae. aeygpti*, midgut and carcass with CHIKV, DENV, WNV, or ZIKV at 1 dpi, 2 dpi, 4 dpi, 7 dpi, or 14 dpi [102,103,104,105]. We focused on significantly downregulated transcripts that were predicted as targets for the significantly upregulated miRNAs in this study. Although no genes that were predicted by all five algorithms were found, our most significantly upregulated miRNAs, miR-9b-5p and miR-989-5p, which had most of their targets predicted by four algorithms, were strongly represented (Table S6). Among the upregulated miRNAs, 80% were paired with downregulated transcripts but only 22% of the downregulated miRNAs were paired with upregulated transcripts. The top 10 most downregulated genes included studies of DENV, WNV, and ZIKV and were from abundances measured early during the course of infection predominately from 1 and 2 dpi. The greatest number of pairings was from transcript abundances measured early during the course of infection at 1, 2, and 4 dpi. 

### 3.5. RRV is a Target of the Ae. aegypti RNAi Response

**Figure 10:**
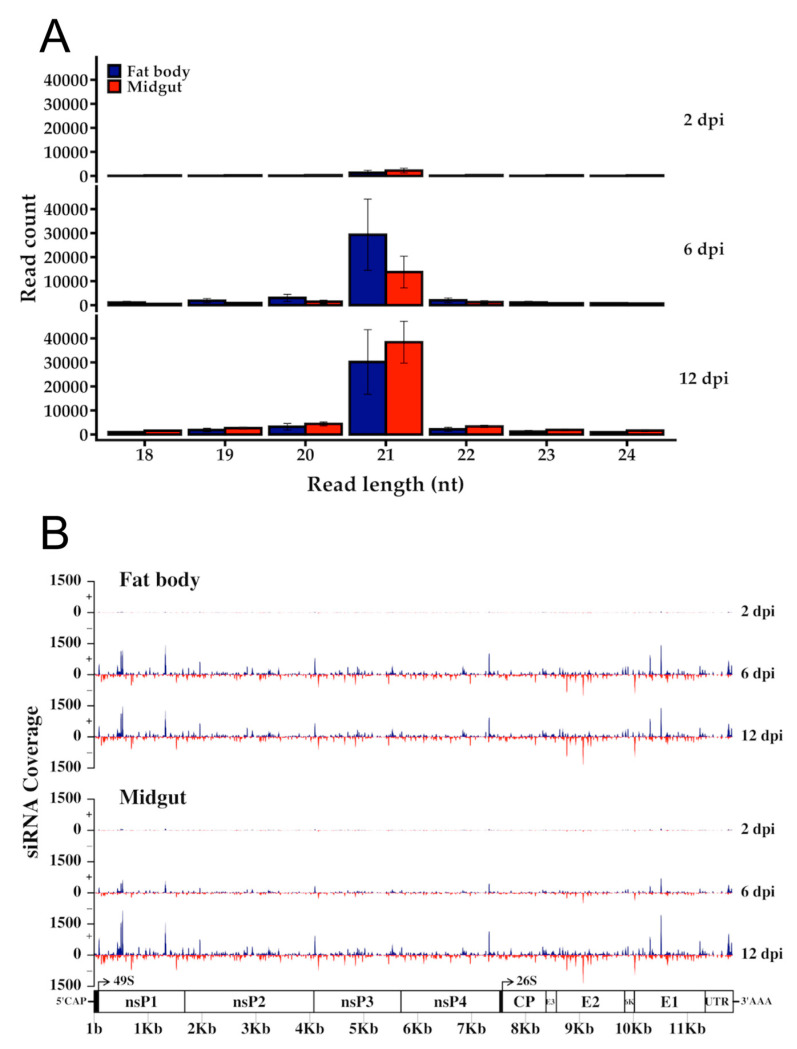
The proliferating 21 nt short interfering RNA (siRNA) count over time was indicative of a progressively increasing viral replication and RNAi response in *Ae. aegypti* mosquitoes. These time-series plots show the (**A**) length distribution of reads mapping to the RRV (T48) genome at 2, 6, and 12 dpi, and (**B**) the 21 nt siRNA coverage of sense (blue lines) and anti-sense (red lines) RRV (T48) genome strands. Error bars represent the SEM of the biological replicates (*N* = 3).

The dsRNA intermediates produced during viral replication become a target of the mosquito RNAi defense response, as reported for a number of alphaviruses including SINV [36,80,106], SFV [39], and CHIKV [31]. The ribonuclease Dicer-2 cleaves these intermediates into 21 nt double-stranded short interfering RNAs (siRNAs), which when bound to the RNA-induced silencing complex (RISC) become a template of the viral RNA sequence and guide for subsequent cleavage. To investigate potential RNAi activity against RRV, we mapped reads to the virus genome that had not aligned with either the *Ae. aegypti* genome or miRBase records. There were 30,652, 238,857, and 420,187 reads in total from days 2, 6, and 12, respectively, which mapped to the viral genome with a length distribution of 15–51 nt. The ratio between reads from fat body and midgut were 1:1.67, 1:0.56, and 1:1.26 for days 2, 6, and 12, respectively. The production of 21 nt sequences, a hallmark of an siRNA response to infection, was evident from day 2, and these increased throughout the time course for both tissues (Figure 10). The distribution of reads mapped somewhat evenly across the genome with a greater number in each case mapping to the sense strand (Figure 10). These results confirm an active RNAi response of *Ae. aegypti* to RRV infection.

### 3.6. Production of RRV-Derived vpiRNAs

In *Ae. aegypti*, there are eight members of the PIWI protein clade (Piwi 1–7 and Ago3) that are involved in piRNA production from transposons, mRNA or viral RNA. The Tudor protein Veneno recruits Yb, Piwi5, and Ago3 [107] to mediate virus-derived piRNA (vpiRNA) production [108] by ping-pong amplification [109], where sense and anti-sense vpiRNA pairs show a nucleotide bias for uridine at position 1 (U_1_) and arginine at position 10 (A_10_), respectively [110,111]. Alphavirus-derived piRNAs have been detected in mosquitoes infected with SFV, CHIKV, ONNV, and SINV [40].

**Figure 11:**
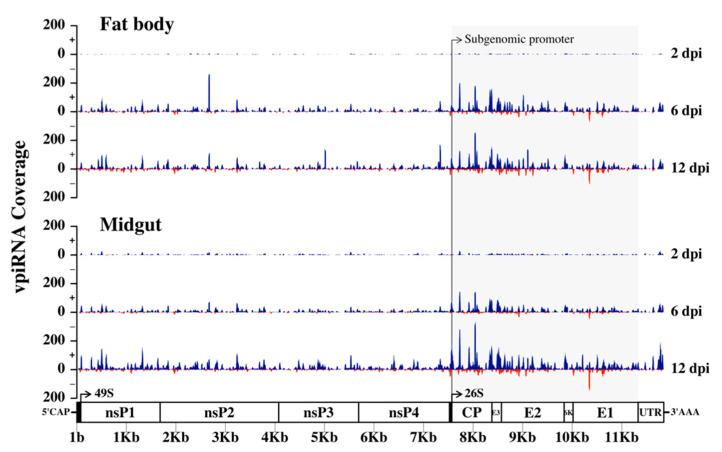
RRV-derived PIWI-interacting RNAs (piRNAs) generated by the fat body and midgut tissues of *Ae. aegypti* mosquitoes. Distribution of 27–29 nt small RNAs that mapped across the sense (blue) and anti-sense (red) strands of the RRV genome at 2, 6, and 12 dpi. Distinct 3’ hotspots indicate virus-derived piRNA (vpiRNA) production against the subgenomic RNA.

To investigate if vpiRNAs are produced in *Ae. aegypti* infected with RRV, we mapped 27–29 nt reads from 2, 6, and 12 dpi to the RRV genome. The number of reads that mapped to the viral genome increased throughout the time course as the infection progressed with a bias for reads mapping to the sense strand (Figure 11). At 6 and 12 dpi for both fat body and midgut samples, there was a moderate clustering of reads at the 3’ end of the genome, which encodes the subgenomic RNA for the production of non-structural proteins (Figure 11). The subgenomic promoters of the alphaviruses SINV, CHIKV, and SFV have been shown to drive subgenomic RNA production in mosquitoes, and in those studies, reads mapped in distinct hotspots to the 3’ non-structural end of the viral genome spanning the subgenomic RNA, and almost exclusively to the sense strand [112]. In our study, clusters of reads were also formed in distinct hotspots spanning the 3’ end of the genome and predominantly mapping to the sense strand. Therefore, it appears that the subgenomic RNA of RRV drives vpiRNA production in *Ae. aegypti*.

**Figure 12:**
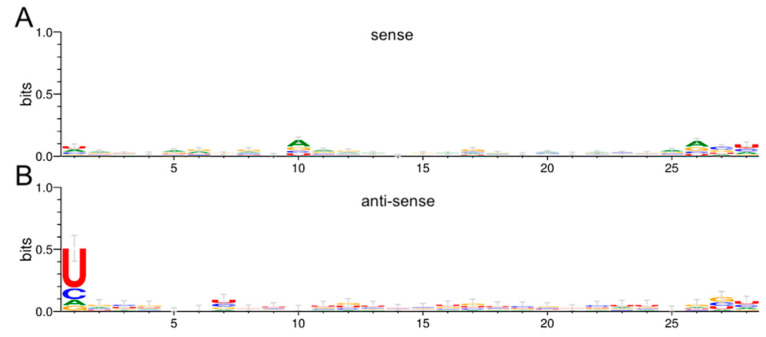
RRV vpiRNA signatures in *Ae. aegypti*. (**A**) There was an A_10_ nucleotide bias, indicative of ping-pong amplification, present in the 28 nt sense vpiRNA-like strands produced in *Ae. aegypti* fat body tissue at 6 dpi with RRV, although there were other biases at positions 1 and 26–28. (**B**) The U_1_ bias, a signature of piRNA ping-pong amplification, was evident in 28 nt piRNAs produced in *Ae. aegypti* fat body at 6 dpi.

To investigate if the vpiRNAs in this study showed the U_1_/A_10_ nucleotide bias characteristic of ping-pong amplification, we used Weblogo software (v3) to determine the nucleotide frequency at each position for reads grouped by the same length, strand, and time-point post-infection. There was a modest A_10_ bias in fat body anti-sense sequences of 28 nt at 6 dpi (Figure 12A), whereas the corresponding U_1_ bias was more readily apparent (Figure 12B).

**Figure 13:**
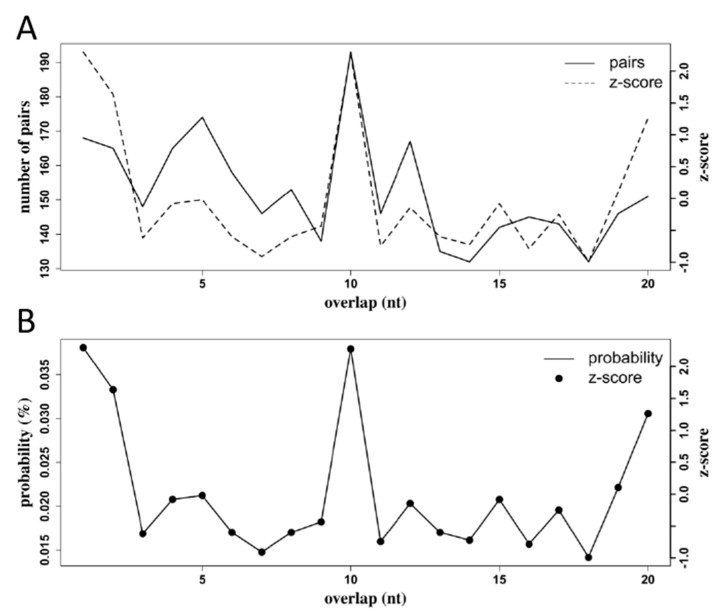
Sequences in the range 27–29 nt from *Ae. aegypti* fat body at 6 days post infection with RRV exhibited a high 10 nt overlap probability. (**A**) A tally of sequence pairs by the number of their overlapping nucleotides, and (**B**) the probability that sequence pairs overlap by the number of intersecting nucleotides. The z-scores (axis on the right) show the mean +/− the number of standard deviations away from the mean. The number of sequences that overlap by 10 nucleotides and the probability that sequence pairs overlap by 10 nt were higher than two standard deviations from the mean. These peaks at 10 nt are a signature of piRNA production by ping-pong amplification.

We used the Small RNA Signatures tool from the Galaxy instance Mississippi.sorbonne-universite.fr to investigate 10 nt overlap probabilities in sequences in the range of 27–29 nt that mapped to the RRV genome. The software pairs sequences with their reverse complement and tally the number of pairs of sequences by the number of their overlapping nucleotides. In fat body tissue at 6 dpi, there was a high frequency of sequence pairs with a 10 nt overlap (Figure 13). The prominent 10 nt peaks for both the number of pairs counted and the probability of seeing pairs with a 10 nt overlap are signatures of piRNA production by ping-pong amplification.

## 4. Conclusions

In summary, we found that RRV promoted the significant differential expression of 14 miRNAs in *Ae. aegypti*, with most occurring in the fat body tissue at 2 dpi. Several genes related to immunity were predicted as targets for the SDE miRNAs from this study, suggesting that RRV may elicit an active defense response by *Ae. aegypti*. A prominent increase in 21 nt sequences was apparent at 6 and 12 days post-infection, indicating a strong siRNA defense response in *Ae. aegypti* infected with RRV. There was a detectable vpiRNA response to RRV in *Ae. aegypti* fat body, and those sequences showed a distinct U_1_ bias, although the A_10_ bias in the corresponding sequences was not as distinct. The overlap probabilities of the 27–29 nt sequences from fat body piRNA-like reads at 6 dpi did show a z-score spike at 10 nt, which provided a complement to the evidence from this study for RRV-derived vpiRNA production in *Ae. aegypti*. This study provides insight into the small RNA responses of *Ae. aegypti* to RRV, and in particular we provide the first characterization of the miRNA responses in *Ae. aegypti* to a previously unexplored old-world alphavirus in this context.

## Figures and Tables

**Table 1:** Differentially expressed *Ae. aegypti* miRNAs upon RRV infection.

Tissue ^1^	dpi	miRNA	Control ^2^	RRV ^2^	FC ^3^	*p*-Value	FDR
FB	2	miR-9b-5p	4752	10,939	2.30	9.10 × 10^−10^	9.55 × 10^−8^
FB	2	miR-317-3p	22,084	13,362	−1.65	4.81 × 10^−6^	2.52 × 10^−4^
FB	2	miR-275-3p	12,465	6290	−1.98	3.74 × 10^−5^	1.31 × 10^−3^
FB	2	miR-275-5p	2208	752	−2.94	4.39 × 10^−4^	9.22 × 10^−3^
FB	2	miR-8-5p	9926	5071	−1.96	2.41 × 10^−4^	6.32 × 10^−3^
FB	2	miR-2945-3p	9070	14,292	1.58	1.40 × 10^−3^	0.02
FB	2	miR-9a-5p	4779	8276	1.73	1.56 × 10^−3^	0.02
FB	2	miR-988-3p	1340	2062	−1.53	1.68 × 10^−3^	0.02
FB	2	miR-34-3p	852	523	−1.63	1.69 × 10^−3^	0.02
FB	2	miR-184-3p	149,602	93,549	−1.60	1.83 × 10^−3^	0.02
FB	2	miR-2940-5p	29,034	16,911	−1.72	1.89 × 10^−3^	0.02
FB	2	miR-989-3p	11,468	67,138	5.85	2.18 × 10^−3^	0.02
FB	12	miR-71-3p	498	366	−2.01	3.85 × 10^−5^	9.58 × 10^−3^
FB	12	miR-2940-3p	11,495	22,203	1.93	4.19 × 10^−4^	0.05
MG	2	miR-9b-5p	9130	15,236	1.66	1.67 × 10^−4^	0.02

^1^ FB: fat body, MG: midgut; ^2^ base mean counts; ^3^ FC: fold change.

## Supplementary Materials

The following are available online at https://www.mdpi.com/1999-4915/12/7/695/s1, Table S1: Primers for RT-qPCR, miScript, and the NGS 3’adapter sequence; Table S2: DESeq2 results of miRNA differential expression; Table S3: miRNA Target prediction; Table S4: OmicsBox Gene Ontology analysis; Table S5: Kyoto Encyclopedia of Genes and Genomes (KEGG) pathways; Table S6: Comparison of transcriptomes.
